# Effect of Lactic Acid Bacteria Strains on the Growth and Aflatoxin Production Potential of *Aspergillus parasiticus*, and Their Ability to Bind Aflatoxin B_1_, Ochratoxin A, and Zearalenone *in vitro*

**DOI:** 10.3389/fmicb.2021.655386

**Published:** 2021-04-22

**Authors:** Cleide Oliveira de Almeida Møller, Luisa Freire, Roice Eliana Rosim, Larissa Pereira Margalho, Celso Fasura Balthazar, Larissa Tuanny Franco, Anderson de Souza Sant’Ana, Carlos Humberto Corassin, Fergal Patrick Rattray, Carlos Augusto Fernandes de Oliveira

**Affiliations:** ^1^Division of Microbiology and Fermentation, Department of Food Science, University of Copenhagen, Frederiksberg, Denmark; ^2^Department of Food Science and Nutrition, Faculty of Food Engineering, University of Campinas, Campinas, Brazil; ^3^Department of Food Engineering, School of Animal Science and Food Engineering, University of São Paulo, Pirassununga, Brazil

**Keywords:** *Lactobacillus*, *Aspergillus*, microbial interaction, mycotoxigenic fungus, mycotoxin inhibition, mycotoxin reduction

## Abstract

The increased consumption of plant-based foods has intensified the concern related to mycotoxin intoxication. This study aimed to investigate the effect of selected lactic acid bacteria (LAB) strains on the growth of *Aspergillus parasiticus* NRRL 2999 and its production of aflatoxin (AF). The ability of the heat-killed (100°C for 1 h) LAB strains to bind aflatoxin M_1_ (AFM_1_) in milk and aflatoxin B_1_ (AFB_1_), ochratoxin A (OTA), and zearalenone (ZEN) in potassium phosphate buffer (PPB) was also evaluated *in vitro*. Ten LAB strains were tested individually, by inoculating them simultaneously with the fungus or after incubation of the fungus for 24 or 48 h at 25°C. Double layer yeast extract sucrose (YES) agar, de Man Rogosa and Sharpe (MRS) agar, and YES broth were incubated for 7 days at 25°C to follow the development of the fungus. *Levilactobacillus* spp. 3QB398 and *Levilactobacillus brevis* 2QB422 strains were able to delay the growth of *A. parasiticus* in YES broth, even when these strains were inoculated 24 h after the fungus. The inhibitory effect of these LAB strains was confirmed by the reduction of fungus colony size, suggesting dominance of LAB by competition (a Lotka-Voltera effect). The production of AFB_1_ by *A. parasiticus* was inhibited when the fungus was inoculated simultaneously with *Lactiplantibacillus plantarum* 3QB361 or *L. plantarum* 3QB350. No AFB_1_ was found when *Levilactobacillus* spp. 2QB383 was present, even when the LAB was inoculated 48 h after the fungus. In binding studies, seven inactivated LAB strains were able to promote a reduction of at least 50% the level of AFB_1_, OTA, and ZEN. This reduction varied depending on the pH of the PPB. In milk, however, only two inactivated LAB strains were able to reduce AFM_1_, with a reduction of 33 and 45% for *Levilactobacillus* spp. 3QB398 (*Levilactobacillus* spp.) and *L. brevis* 2QB422, respectively. Nevertheless, these results clearly indicate the potential of using LAB for mycotoxin reduction.

## Introduction

An important food safety concern is the presence of natural contaminants such as the mycotoxins (Schatzmayr and Streit, 2013; Eskola et al., 2020; Baazeem et al., 2021). In 2019, mycotoxins were reported to be the main hazard in food products at EU borders (RASFF, 2020). As recently as 2020, a survey of global food crop contamination reported an incidence of 25% of mycotoxins above the EU and Codex limits (Eskola et al., 2020). The potential toxic effects of mycotoxins including immunosuppression and carcinogenic effects have stimulated considerable research efforts aiming at reducing the mycotoxin levels in foods (Corassin et al., 2013; Oliveira et al., 2014; Gummadidala et al., 2019; Cuevas-González et al., 2020; Gao et al., 2020). In particular, the aflatoxins (AFs) B_1_ (AFB_1_) and M_1_ (AFM_1_) were classified in Group 1 (human carcinogen) by the International Agency for Research on Cancer (2012), while ochratoxin A (OTA) was categorized in Group 2B, probable carcinogen to humans (International Agency for Research on Cancer, 1993). The incidence of AFM_1_ in milk is of concern, because milk is the main nutritional source for infants and children (Conteçotto et al., 2021). AFM_1_ in the milk is derived from the biotransformation of AFB_1_ by the cow (Barnes, 1970; Galvano et al., 1996; Pauletto et al., 2020). Since AFM_1_ binds to the casein in the milk, it may be carried into high-content protein products such as cheeses, and this is worrying considering the highly consumption of cheeses by people of all ages (Khaneghah et al., 2021). Considering the health risks posed by AFM_1_, several countries have set maximum permissible levels (MPL) for this toxin in milk. The European Union and the United States Food and Drug Administration (FDA) have set MPLs of 0.05 and 0.5 μg L^−1^ for AFM_1_ in milk, respectively (Oliveira et al., 2014).

Many genera of molds have the ability to produce mycotoxins (Campagnollo et al., 2020). The main genera of mycotoxin-producing fungi include *Aspergillus*, *Penicillium*, *Fusarium*, and *Alternaria* (Egbuta et al., 2017). The mycotoxins produced by these genera include the AFs, fumonisins (FBs), OTA, zearalenone (ZEN), and trichothecenes such as deoxynivalenol (DON; Palumbo et al., 2020). Levels of these mycotoxins in food are regulated in many countries because of their frequent occurrence and their high toxigenic potential (Oliveira et al., 2014; Franco et al., 2019). The main food commodities produced worldwide and associated with mycotoxin contamination are mainly cereals, seeds, nuts, fruit, vegetables, herbs, and spices (RASFF, 2020).

The present situation clearly shows that mycotoxin occurrence is an increasing worldwide problem despite the many developments of good agricultural and manufacturing practices in the food chain. The impact of mycotoxins on human and animal health, as well as on economic losses is significant (Eskola et al., 2020). According to a recent estimation (World Health Organization, 2016), the mycotoxin contamination problem is especially evident in developing countries, where 500 million people are exposed to mycotoxins, and 160 million children under the age of five suffer from stunting associated to exposure to these toxins. Besides the high cost related to research and regulatory activities, the yearly direct impact associated with mycotoxins in food and feed production is immense, with the extent varying according to the region and the weather (Gbashi et al., 2018; Agriopoulou et al., 2020). In the United States, the aflatoxin-associated losses in food and feed crops are estimated to be between 300 million and 1.7 billion USD per year (Pitt and Miller, 2017). While 900 million USD per year are the total social costs of aflatoxin estimated to the Philippines, Thailand, and India (European Commission, 2016). The annual cost related to crops contaminated with aflatoxins in Africa is estimated to be between 670 and 750 million USD (Gbashi et al., 2018). The detailed data related to economic impact in the EU are not reported. Avoidance of mycotoxigenic fungi in the field is of extreme importance as the level of mycotoxins in the plants increases due to the environmental conditions (temperature and humidity) and management practices, especially during storage after harvest. Since mycotoxins are stable and cannot be reduced during processing, they end up in the crops used in the production of plant-based foods (Bullerman and Bianchini, 2007; Cinar and Onbaşı, 2019). Natural solutions have been proposed to prevent mycotoxin formation and to degrade or avoid the bioavailability of mycotoxins in foods (Avantaggiato et al., 2014; Agriopoulou et al., 2020; Muhialdin et al., 2020). Efforts to investigate the suitability of lactic acid bacteria (LAB) have been proposed since the 1960s (Ciegler et al., 1966), but the potential of these bacteria to reduce the risk associated with mycotoxins is still at an early research stage. In this context, three main mechanisms have been proposed for mycotoxin reduction by LAB: (i) binding of the mycotoxin to the bacterial cell wall components, (ii) degradation of the mycotoxins into less toxic substances by the LAB, or (iii) inhibition of the mold growth or aflatoxin biosynthesis by specific bacterial metabolites (Ben Taheur et al., 2019a).

Many LAB are regarded by the FDA and general recognized as safe (GRAS), while the European Food Safety Authority (EFSA) applies the qualified presumption of safety (QPS) principle (EFSA Panel on Biological Hazards (BIOHAZ) et al., 2017; FDA, 2018; Hill et al., 2018). Their nature of non-pathogenicity and non-toxicity allows many applications of LAB with the purpose of improving safety and quality in food processing. In a recent study, Gonçalves et al. (2020) successfully used heat-killed cells of LAB (*Lacticaseibacillus rhamnosus* and *Lactococcus lactis*) and *Saccharomyces cerevisiae*, alone or in combination, for binding up to 100% of AFM_1_ in cheese. Furthermore, a *Lentilactobacillus kefiri* strain was able to reduce 80–100% of AFB_1_, OTA, and ZEN through binding in milk (Ben Taheur et al., 2017). Despite the reported positive effect of LAB in the reduction of mycotoxins, the usefulness of LAB it is still uncertain when the level of mycotoxins is high and well-established in the food matrix (Ben Taheur et al., 2019a). Intracellular and extracellular extracts of microorganisms, like *Pediococcus parvulus*, *Bacillus subtilis*, and *Candida utilis* have proven to be important sources of enzymes that are able to biotransform some of the well-known mycotoxins, such as OTA (Abrunhosa et al., 2014), ZEN, and AFB_1_ (Huang et al., 2018). Various pathways have been proposed for mycotoxin biodegradation (Zhang et al., 2018; Ben Taheur et al., 2019a).

The purpose of this work was to screen the ability of the selected LAB strains to inhibit growth of *A. parasiticus* and to reduce the formation of AFB_1_, AFB_2_, AFG_1_, and AFG_2_. In addition, the ability of the strains to reduce the levels of the mycotoxins in spiked buffer models (AFB_1_, OTA, and ZEN) and milk (AFM_1_) was examined.

## Materials and Methods

### Lactic Acid Bacteria Strains

Ten LAB isolates from Brazilian artisanal cheeses (isolation conditions indicated in Table 1) were selected; four strains of *Lactiplantibacillus plantarum* (formerly *Lactobacillus plantarum*, comprising strains 1QB147, 1QB314, 3QB350, and QB361), two strains of *Levilactobacillus brevis* (formerly *Lactobacillus brevis*, comprising strains 2QB422 and 3QB446), and four strains of *Levilactobacillus* spp. (formerly *Lactobacillus* spp., comprising strains 1QB4593, 2QB383, 3QB167, and QB398). The identity of the isolates was confirmed using the MALDI-TOF MS Biotyper by Margalho et al. (2020).

**Table 1:** Identification and source of lactic acid bacteria (LAB) isolates.

LAB species	Code	Cheese type	City/State	Region	Microregion
*Lactiplantibacillus plantarum*	1QB147	Coalho	Cajazeiras – PB	Northeast	-
*Lactiplantibacillus plantarum*	1QB314	Colonial	Lacerdópolis – SC	South	-
*Lactiplantibacillus plantarum*	3QB350	Caipira	Jaraguari – MS	Center-West	-
*Lactiplantibacillus plantarum*	3QB361	Minas artesanal	São João del Rei – MG	Southeast	Campo das Vertentes
*Levilactobacillus brevis*	2QB422	Caipira	Ribas do Rio Pardo – MS	Center-West	–
*Levilactobacillus brevis*	2QB446	Caipira	Anhanduí – MS	Center-West	–
*Levilactobacillus* spp.	1QB459	Caipira	Jaraguari – MS	Center-West	–
*Levilactobacillus* spp.	2QB383	Minas artesanal	São João del Rei – MG	Southeast	Campo das Vertentes
*Levilactobacillus* spp.	3QB167	Manteiga	Cajazeiras – PB	Northeast	–
*Levilactobacillus* spp.	3QB398	Minas artesanal	Medeiros – MG	Southeast	Canastra

Source: Margalho et al. (2020).

### Growth Experiments

#### Preparation of LAB Inoculum

Inocula of the 10 strains were prepared according to Møller et al. (2021). Briefly, 100 μl of each of the thawed frozen stocks (−80°C, in 20% glycerol) were individually inoculated into 10 ml MRS broth, and incubated at 30°C in a Biological Oxygen Demand (BOD) incubator (Tecnal, Sao Paulo, Brazil) for 24 h, at static condition in the dark. The grown culture was centrifuged (6,000 × *g* for 10 min at 4°C), and the cell pellet was resuspended in 10 ml 0.9% NaCl and centrifuged (6,000 × *g* for 10 min at 4°C). The washed, resuspended culture was adjusted to 0.5 MCFarland (~ 10^8^ CFU/ml) and diluted in 0.9% NaCl in order to obtain a final cell density of 5.0 log_10_ CFU g^−1^ (checked by plate count).

#### Preparation of the Conidial Suspension

*Aspergillus parasiticus* NRRL 2999 (a strain with the ability to produce mycotoxins such as AFB_1_, AFB_2_, AFG_1_, and AFG_2_) was kindly donated by the United States Department of Agriculture. This strain was isolated from soil in Uganda as reported by Singh and Cotty (2019). The strain was prepared according to Wigmann et al. (2016) by inoculating it in Malt Extract Agar (MEA; Acumedia, Lansing, Michigan, United States; Malt Extract: 20.0 g; Peptone: 1.0 g; Glucose: 30.0 g; Agar: 20 g; and Distilled Water: 1 L) and incubating it aerobically at 25°C in a BOD incubator for 5 days, at static condition in the dark. Conidia were collected by scraping the mycelium from each plate with sterile distilled water containing 0.1% Tween 80 (Labsynth, Diadema, Brazil). Subsequently, they were filtered and then centrifuged at 11,000 g three consecutive times for 15 min at 4°C (Sorvall Legend XTR, Thermo Scientific, Hampton, Unites States). The final concentration of conidia in the fungal suspension was determined using a Neubauer chamber (Sigma-Aldrich, Darmstadt, Germany). The conidial suspension was diluted and used to reach a final concentration of 5.0 log_10_ cfu ml^−1^ in the growth trials (checked by plate count).

#### Incubation of LAB With *A. parasiticus* in Broth

The method was adapted from Bouillon et al. (2019) and performed in 200 μl YES broth using 96-well microtiter plates. Negative control (50 μl of non-inoculated 0.9% saline), positive control of the fungus (25 μl of the fungus suspension plus 25 μl of 0.9% saline), and positive control of each LAB (25 μl inoculum of the individual LAB strain plus 25 μl of 0.9% saline) were added to 200 μl YES broth. When the fungus was tested in combination with the respective LAB strain, a 25 μl inoculum of each microorganism was added to 200 μl YES broth, and a final volume of 250 μl was reached. The final concentration was 5.0 log_10_ cfu ml^−1^ for the LAB and/or 5.0 log_10_ spores ml^−1^ for the fungus. Three independent trials were performed in triplicate. The microtiter plates were incubated at 25°C in a BOD incubator (Tecnal, Sao Paulo, Brazil) for 7 days, at static condition in the dark. Growth of the fungus in the YES broth was evaluated after incubation by visual inspection of the microplates. The treatments were compared relatively, and the effect of each tested combination was ranked in relation to the fungus positive control. Symbols were assigned according to the effect of each LAB in comparison with the growth of *A. parasiticus* alone (positive control of the fungus). (+++) indicates the maximum effect on growth without total inhibition of the fungus growth by LAB and (+) indicates very little effect of LAB on growth of the fungus.

#### Incubation of LAB With *A. parasiticus* Using an Agar Plate Technique

A double-layer agar technique was adapted from Aunsbjerg et al. (2015) and applied in triplicate using petri dishes (8.5 cm round shape). One milliliter inoculum of the individual LAB strains (final concentration of 5 log_10_ cfu mL^−1^) was covered with 10 ml MRS agar, which when solidified was covered with 10 ml YES Agar (Samson et al., 2002). Ten microliters of the fungus suspension (final concentration of 5 log_10_ spores ml^−1^) were drop plated into the center of the plate of the solidified YES agar. The development of the fungus was followed by measuring the diameter of fungal growth every 24 h over a 7 days incubation period at 25°C. Plates were incubated in a BOD incubator (Tecnal, Sao Paulo, Brazil), at static condition in the dark.

#### Inhibition of *A. parasiticus* Growth and Mycotoxins Production in Broth

The 10 LAB strains were applied individually at the level of 5.0 log_10_ cfu ml^−1^ at each of the following three inoculum time-points to the YES broth (10 ml): (a) simultaneously with the *A. parasiticus* (5.0 log_10_ spores ml^−1^); (b) after 24 h of incubation of the *A. parasiticus*; or (c) after 48 h of incubation of the *A. parasiticus*. All incubations were performed at 25°C, at static condition in the dark. Samples (1 ml) for mycotoxin determination were taken just after inoculation with *A. parasiticus* alone (control) or combined with the tested LAB strain. Further 1 ml samples were taken 7 days after the inoculation of the fungus. Samples were dispersed in Eppendorf tubes, centrifuged at 3,000 × *g* for 15 min, and the supernatants were kept at −20°C until analysis. Samples were prepared by diluting 20 μl of supernatants in 2 ml of milli-Q water (Millipore, Burlington, MA, United States). Then, 100 μl of the diluted supernatant were mixed with 900 μl acetonitrile:water (50:50), and 500 μl were transferred to a new Eppendorf tube containing 200 μl hexane and 100 μl trifluoroacetic acid for derivatization of AFB_1_ and AFG_1_ (Oliveira et al., 2008). The mixture was kept at 35°C for 10 min, then evaporated to near-dryness, and diluted in 500 μl acetonitrile:water (50:50). Final extracts were filtered through a 0.45 μm PTFE membrane and subjected to AF determination using HPLC according to Oliveira et al. (2008). Triplicate samples were analyzed by injecting 20 μl of sample with an isocratic elution by water:acetonitrile:methanol (60:20:20) for a run time of 10 min (flow rate of 1.0 ml min^−1^) in a Shimadzu 10VP liquid chromatograph (Kyoto, Japan) with a 10 AXL fluorescence detector (excitation at 360 nm and emission above 440 nm). A Kinetex C_18_ column (Phenomenex, Torrance, CA, United States) 4.6 × 150 mm, 2.6 μm particle size and an in-line filter of 0.5 μm were used. The limit of detection (LOD) and the limit of quantification (LOQ) were calculated from matrix matched calibration curves using signal to noise ratios of 3 and 10, respectively. Under these conditions, the LOD and LOQ values for each aflatoxin (AFB_1_, AFB_2_, AFG_1_, or AFG_2_) were 0.1 and 0.3 μg ml^−1^, respectively.

### Testing the Reduction of Mycotoxins by the Binding Ability of LAB

#### Preparing the LAB Cells

The LAB strains were also investigated for their potential to reduce levels of mycotoxins in spiked buffer and milk. Preparation of the cells was performed according to Bovo et al. (2013). The LAB inocula were obtained as described in section “Preparation of the conidial suspension” (before washing steps). The 1 ml bacterial suspensions (9.0 log_10_ cfu ml^−1^) were dispersed in Eppendorf tubes, centrifuged at 3,000 × *g* for 15 min, and the cell pellets exposed to 100°C for 1 h. The heat-treated cell pellets were then washed twice with 0.9% NaCl and kept at −20°C until execution of the binding studies.

#### Binding Studies in Milk Spiked With Aflatoxin M_1_

One milliliter skimmed milk spiked with AFM_1_ at the level of 500 ng L^−1^ was added to the bacterial cell pellet (obtained from 1 ml of the 9.0 log_10_ cfu ml^−1^ bacterial suspension, inactivated as described in section “Preparing the LAB cells”) and incubated at 37°C for 15 min (Ismail et al., 2017). Negative controls (milk + LAB cells) and positive controls (milk + AFM_1_) were also prepared. All incubations were performed in triplicate. After incubation, the cells were removed by centrifugation (3,000 × *g* for 15 min) and the supernatant was collected for quantification of the remaining AFM_1_.

#### Binding Studies in Buffer Spiked With AFB_1_, OTA, and ZEN

One milliliter samples of 0.1 M potassium phosphate buffer (PPB) with different pH values (3.0 or 6.5) and spiked with AFB_1_, OTA (at the level of 1.0 μg ml^−1^), and ZEN (2.0 μg ml^−1^), were added to the bacterial cells pellet (obtained from 1 ml of the 9.0 log_10_ cfu ml^−1^ bacterial suspension, inactivated as described in section “Preparing the LAB cells”) and incubated at 37°C for 5 as well as for 15 min (Campagnollo et al., 2015). Negative controls (PPB + LAB cells) and positive controls (PPB + mycotoxin mixture) were also prepared. All incubations were performed in triplicate. After incubation, the cells were removed by centrifugation at 3,000 × *g* and the supernatants were collected for quantification of remaining AFB_1_, OTA, and ZEN for each treatment.

#### Quantification of Mycotoxins

Aflatoxin M_1_ was extracted from supernatants of spiked milk samples as described by Flores-Flores and González-Peñas (2017). Supernatants from spiked buffer samples were individually diluted in acetonitrile (100 μl in 10 ml), and after filtration (0.22 μm) were again diluted in acetonitrile (100 μl in 1.0 ml), and then collected for quantification of AFB_1_, OTA, and ZEN according to Franco et al. (2019). Five microliter of each final extract from milk samples or diluted buffer samples were injected into a Waters Acquity I-Class system (Waters, Milford, MA, United States) equipped with a BEH C18 column (2.1 × 50 mm, 1.7 μm) and coupled to a Xevo TQ-S® mass spectrometer (Waters, Milford, MA, United States). The chromatographic conditions were as described by Franco et al. (2019), with a flow rate of 0.5 ml min^−1^ (total chromatographic run time of 10 min). MS/MS parameters were as stated in Table 2.

**Table 2:** LC-MS/MS conditions for investigating the levels of aflatoxin (AF) M1 (AFM_1_) in skimmed spiked milk (5.0 ng L^−1^), as well as aflatoxin B1 (AFB_1_) ochatoxin A (OTA), and zearalenone (ZEN) in potassium phosphate buffer (PPB) spiked samples (5.0 ng L^−1^), after treatment with LAB heat inactivated cells.

Mycotoxin ID	Retention time (min)	Mass (g/mol)	Molecular ion	Transition (m/z)	Cone voltage (V)	Colision energy (V)	Range of calibration curve (μg ml^−1^)	LOD (μg ml^−1^)	LOQ (μg ml^−1^)
AFM_1_^*^	4.03	328.3	[M + H]^+^	329.0 > 273.1^a^	52	24	0.05–0.5	0.01	0.05
				329.0 > 243.0^b^	52	38			
AFB_1_^**^	4.80	312.3	[M + H]^+^	312.7 > 284.9^a^	94	36	1.0–50.0	0.5	1.0
				312.7 > 241.1^b^	94	22			
OTA^**^	5.99	403.1	[M + H]^+^	404.0 > 238.9^a^	35	22	1.0–50.0	0.7	1.1
				404.0 > 357.9^b^	35	12			
ZEN^**^	5.98	318.1	[M − H]^−^	317.1 > 175.1^a^	50	23	1.0–100.0	0.23	0.6
				317.1 > 130.9^b^	50	33			

a Transitions used for quantification.

b Transitions used for confirmation.

* AFM_1_ standards for calibration curve: 0, 0.0625, 0.125, 0.25, and 0.5 ng ml^−1^, according to Corassin et al. (2013).

** Mycotoxins standards (mix): AFB_1_: 1.25, 2.5, 5, and 10 ng ml^−1^; OTA: 1.25, 2.5, 5, and 10 ng ml^−1^; and ZEN: 2.5, 5, 10, and 20 ng ml^−1^, according to Franco et al. (2019).

### Data Analysis

The growth of the fungus was modeled by applying the adapted version (Equation 1) of the primary model developed by Baranyi et al. (1993) and Dantigny et al. (2005), to allow accessing of the effect of LAB on growth of the mycotoxigenic fungus.

$$r\;\left(\mathrm{cm}\right)=\mu \left(t-\lambda \right)$$ (1)

Where *r* is the colony diameter (cm) as a function of time (*t*), *μ* is the multiplication rate (cm day^−1^), and *λ* is the time obtained by the intersection (days).

Normality test was performed to verify variability in the samples, the Friedman test was applied due to a not normal distribution of data, and evaluation of the differences in the kinetic parameters of the *A. parasiticus* was performed according to the approach suggested by Ferreira (2011).

The effect of LAB on inhibition of mycotoxins production was evaluated by comparing median, minimum, and maximum values of each treatment (performed in triplicate) to the levels of mycotoxins produced by the non-treated fungus in YES. Medians of AFs levels were compared using Mood Median test of XLStat software (version 2020.1.1) at 5% significance level (Addinsoft, 2021).

The reduction of mycotoxins after the treatment of spiked “*in vitro*” models and milk, performed in triplicate, were subjected to ANOVA using the General Linear Model of SAS® (SAS Institute, 1992). When applicable, means showing significant differences were compared using the Fisher protected least significant difference test, considering the 0.05 level of probability.

## Results and Discussion

### Effect of Lactic Acid Bacteria on Growth of Mycotoxigenic *Aspergillus parasiticus*

**Figure 1:**
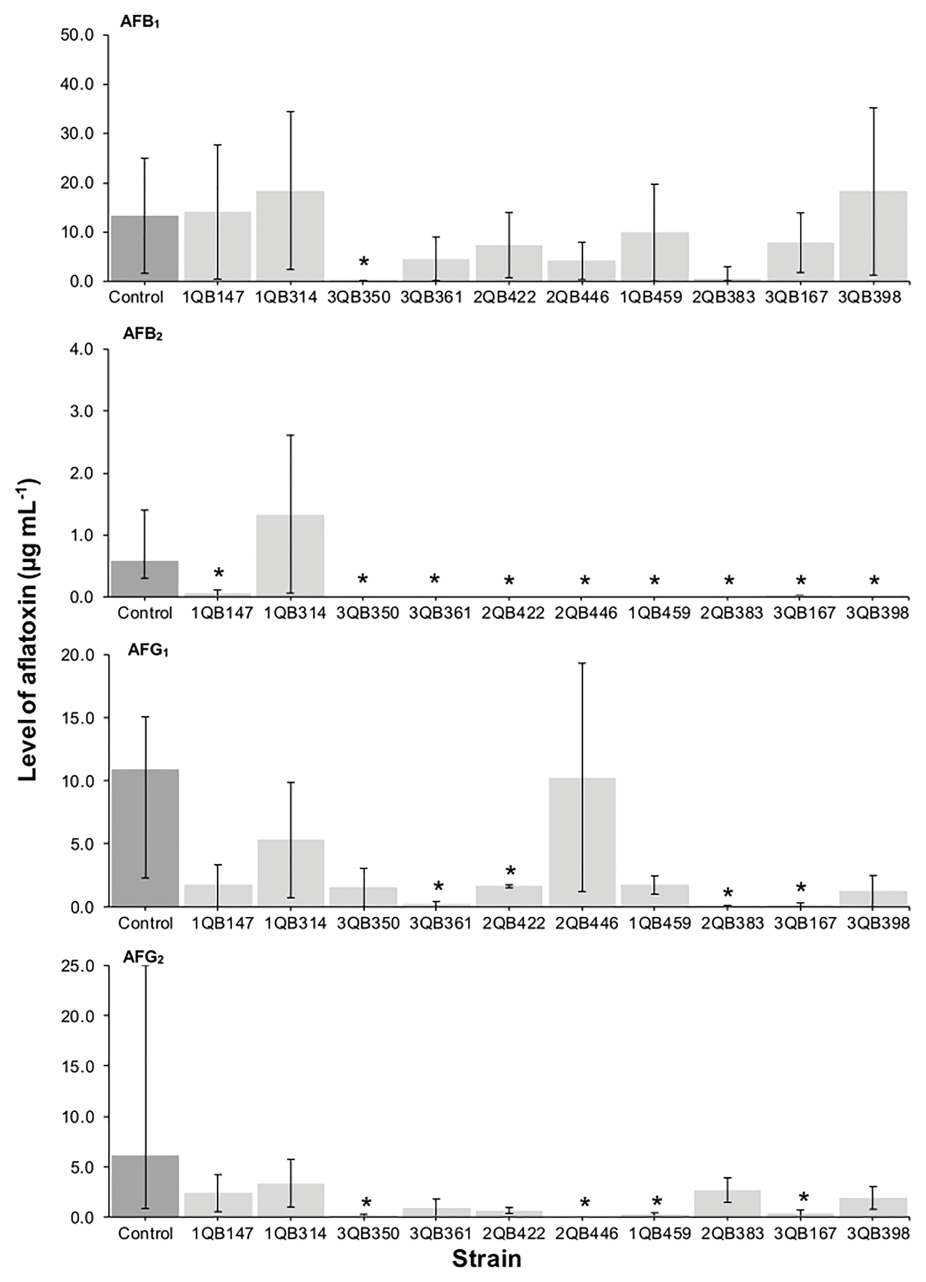
Effect of LAB on production of aflatoxins B_1_ (AFB_1_), B_2_ (AFB_2_), G_1_ (AFG_1_), and G_2_ (AFG_2_), by *A. parasiticus* NRRL 2999 in co-cultured YES broth after 7 days of incubation at 25°C. LAB was inoculated (5.0 log_10_ cfu ml^−1^) simultaneously with the fungus (Inoculation = T 0 h). The individually tested LAB included: four strains of *Lactiplantibacillus plantarum* (former *Lactobacillus plantarum*, comprising strains 1QB147, 1QB314, 3QB350, and QB361), two strains of *Levilactobacillus brevis* (former *Lactobacillus brevis*, comprising strains 2QB422 and 3QB446), and four strains of *Levilactobacillus* spp. (former *Lactobacillus* sp., comprising strains 1QB4593, 2QB383, 3QB167, and QB398). Results are expressed as median values of three independent experiments, with minimum and maximum values indicated as error bars. ^*^Median values with asterisks differ significantly from controls (*p* < 0.05).

**Figure 2:**
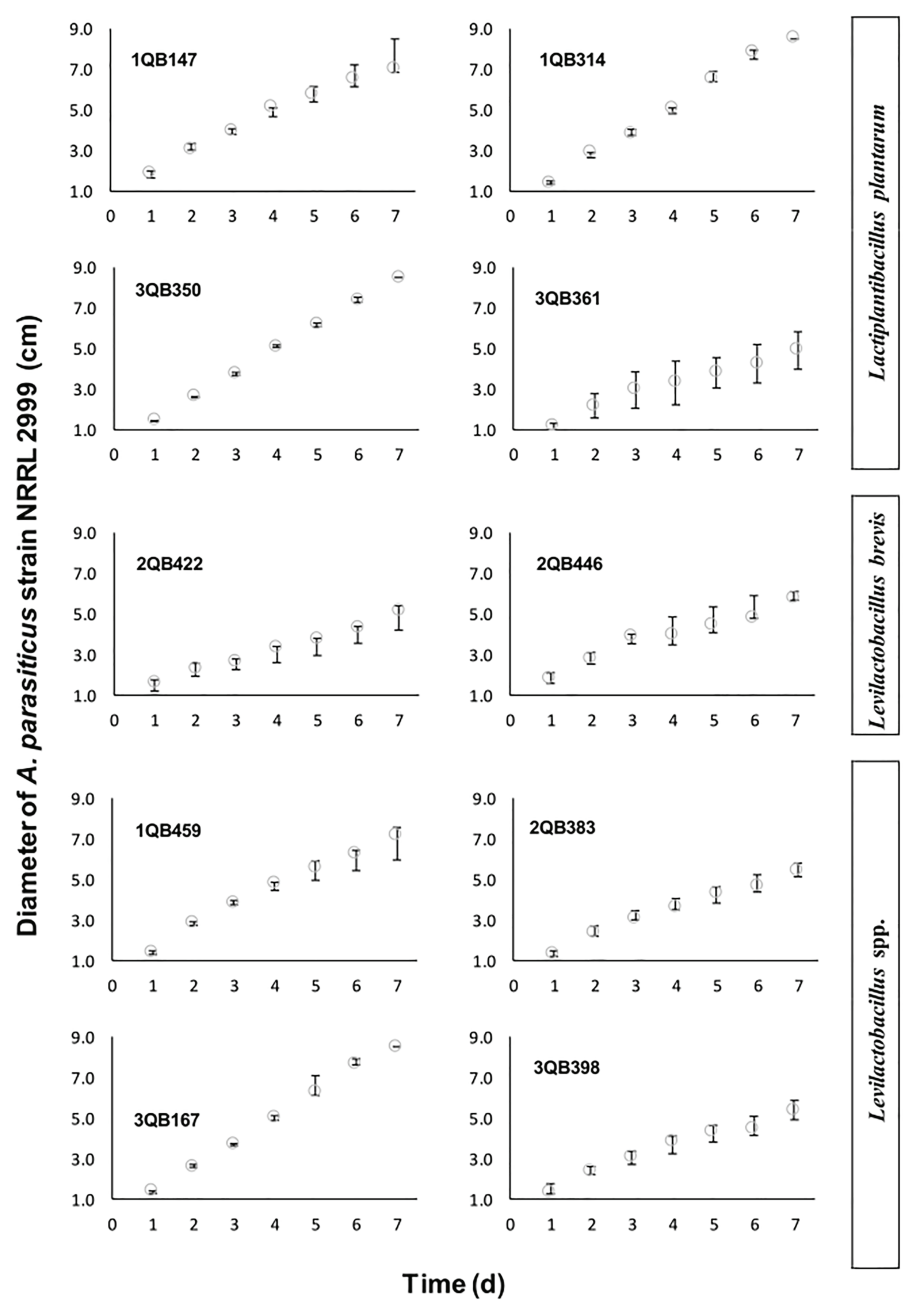
Diameter increase of *A. parasiticus* NRRL 2999 drop plated (inoculated at the level of 10^5^ log_10_ cfu ml^−1^) on the center of the YES agar, and incubated for 7 days at 25°C. The YES agar was covering a layer of MRS agar pour plated with one of the tested LAB (5.0 log_10_ cfu ml^−1^), and included: four strains of *Lactiplantibacillus plantarum* (former *Lactobacillus plantarum*, comprising strains 1QB147, 1QB314, 3QB350, and QB361), two strains of *Levilactobacillus brevis* (former *Lactobacillus brevis*, comprising strains 2QB422 and 3QB446), and four strains of *Levilactobacillus* spp. (former *Lactobacillus* sp., comprising strains 1QB4593, 2QB383, 3QB167, and QB398). Results are expressed as median values of three independent experiments, with minimum and maximum values indicated as error bars.

The screening of LAB performed in microtiter plate (250 μl) was not conclusive but it gave an indication that all tested LAB strains had an impact on the growth of the fungus (Table 3). While strains 1QB459 (*Levilactobacillus* spp.), 1QB314 (*L. plantarum*), and 2QB446 (*L. brevis*) had a greater impact (+++, Table 3) on growth of *A. parasiticus*, a lower impact was seen for strains 3QB398 (*Levilactobacillus* spp.), 3QB167 (*Levilactobacillus* spp.), and 3QB361 (*L. plantarum*; +, Table 3). By scaling-up the assay to 10 ml (in test tubes), it was confirmed that strain 2QB422 (*L. brevis*; Table 3) had the potential to reduce the fungal growth not only when inoculated simultaneously with the fungus but also when it was inoculated at 24 and 48 h after the fungus was incubated. This finding indicates the potential of the strain to inhibit the growth of the fungus, even after the adaptation phase of the fungus to the environment. A possible explanation of the fungal growth inhibition could be the production of antimicrobial peptides, as reported in the literature (Dalié et al., 2010; Muhialdin et al., 2011; Hashemi and Gholamhosseinpour, 2019). While strain 3QB398 (*Levilactobacillus* spp.) was shown to have inhibition activity against the growth of the fungus at earlier stages of the fungus incubation (0 and 24 h), strains 3QB350 (*L. plantarum*) and 1QB314 (*L. plantarum*) were effective in reducing growth when inoculated at the later stages (24 and 48 h) after the incubation of the fungus. It is speculated that while the mechanism of inhibition of strain 3QB398 (*Levilactobacillus* spp.) may be related to fast production of metabolites in high concentration during growth of the strain, the mechanism of inhibition for strains 3QB350 (*L. plantarum*) and 1QB314 (*L. plantarum*) may be due to the slower production of metabolites as well as lower toxicity of the compounds, or even competition for nutrients. These mechanisms of action of LAB have been reported by Ben Said et al. (2019). Furthermore, they also highlighted the capacity of some LAB to adhere strongly to surfaces and survive there for long periods of time. Margalho et al. (2020) investigated the bacteriocin-forming ability of the LAB strains tested in the present study. The bacteriocin-forming ability may be related to the inhibition mechanism of LAB strains such as the strain with the greatest impact on growth of *A. parasiticus*, which in our study was shown to be *L. brevis* 2QB422 in all stages of application (Table 3). This strain was also found to be positive for bacteriocin production and effective against different strains of *Listeria monocytogenes* (Margalho et al., 2020). Similar to our work, Ghanbari et al. (2018) observed that *L. plantarum* and *Lactobacillus delbrueckii* subsp. *lactis* significantly reduced the mycelial growth of *A. parasiticus*. Luz et al. (2017) also described antifungal activities of *L. plantarum CECT 749* against *A. parasiticus* in potato dextrose agar (PDA), while no activity was observed with *Lb. delbrueckii* subsp. *bulgaricus CECT 4005*. Also in agreement with the present study, Poornachandra Rao et al. (2019) showed that a cell-free supernatant of *L. plantarum MYS44* inhibited *A. parasiticus* through general morphological changes, such as destruction of the hyphae wall and inhibition of the germination of the conidia. Strains of *Lacticaseibacillus casei* and *Lactobacillus acidophilus* have also shown to have inhibition effects against *A. parasiticus* (Abbaszadeh et al., 2015). LAB have been reported to produce compounds during growth with potent antifungal effects which may damage the fungal membrane and cell wall structure, resulting in lysis of hyphae and spores (de Souza de Azevedo et al., 2020; Li et al., 2021). As indicated by the two first assays, *L. brevis* 2QB422 was the most effective strain in suppressing the growth of *A. parasiticus*, which reached a maximum diameter of 4.91 ± 0.6 cm (Table 3; Figure 1), followed by the range of 5.26–5.46 cm for strains 3QB361 (*Lactiplantibacillus plantarum*), 3QB398 (*Levilactobacillus* spp.), 2QB383 (*Levilactobacillus* spp.), and 2QB446 (*L. brevis*). In order to clarify the full inhibition potential of the tested LAB strains on the fungus, the diameter was followed daily for 7 days at 25°C (Figure 2). The parameters obtained for the growth rate of the fungus (Table 3), also showed the same effect when comparing the maximum growth reached after 7 days incubation. Since growth of LAB was much faster and the maximum population density was reached at earlier stages of the fungus growth, microbial interaction modeling was not possible. However, it is clear that inhibition was not related to the Jameson effect, which describes the phenomena of the dominant microorganism reaching the maximum population and completely interrupting the growth phase of the other population (Jameson, 1962). In fact, the growth patterns shown in the present study indicate a Lotka-Volterra competition (Lotka, 1956), which describes the phenomena of the dominant microorganism reaching the maximum population and lowering the growth rate of the other microorganism (Table 3). While growth rates below 1.0 cm day^−1^ were reached by treatment with strains having inhibiting activity against *A. parasiticus* growth, rates equal and higher than 1.0 cm day^−1^ were reached when no effect of the strains on the growth of the fungus was shown. Even though the LAB strains were not able to completely suppress the growth of the *A. parasiticus*, it is clear that they have the potential to extend the shelf life and possibly improve safety of food matrices contaminated with this fungus. Studies in regional cheeses where the LAB strains were isolated are rare and Margalho et al. (2020) are one of the first investigations already reported, which highlights the importance of the present study. Interactions of LAB with mycotoxigenic aspergilli have been proposed to be good examples of biological control in the field. However the need for more research to deeper understand the mechanisms and to develop novel biocontrol technologies remains to be explored (Pócsi et al., 2020).

**Table 3:** Effect of LAB on the growth of mycotoxigenic *Aspergillus parasiticus* NRRL 2999, investigated in yeast extract sucrose (YES) broth and double layer agar plates with de Man Rogosa and Sharpe (MRS) and YES agars.

LAB strains	LAB and Fungus co-cultured in						
	YES broth				Double layer in MRS-YES agars		
	Growth of fungus inoculated simultaneously with LAB in microplate assay (250 μl) ^α^	Growth of fungus in test tube (10 ml) inoculated with LAB at different stages ^β^ of fungus incubation (h) at 25°C ^γ^			Parameter estimates from the fitting of fungus growth on double agar plate, inoculated with LAB and incubated at 25°C for 7 days		
		0	24	48	Growth rate^*^ μmax (cm day^−1^)	Maximum^**^ diameter (cm)	*R*^2^
*Lactiplantibacillus plantarum* 1QB147	++	*X*			1.00 ± 0.1	7.45 ± 0.9	0.99
*Lactiplantibacillus plantarum* 1QB314	+++		*X*	*X*	1.20 ± 0.0	8.50 ± 0.0	0.99
*Lactiplantibacillus plantarum* 3QB350	++		*X*	*X*	1.20 ± 0.0	8.50 ± 0.0	0.99
*Lactiplantibacillus plantarum* 3QB361	+	*X*		*X*	0.70 ± 0.1	5.26 ± 0.6	0.96
*Levilactobacillus brevis* 2QB422	++	*X*	*X*	*X*	0.60 ± 0.1	4.91 ± 0.6	0.96
*Levilactobacillus brevis* 2QB446	+++			*X*	0.73 ± 0.1	5.85 ± 0.2	0.93
*Levilactobacillus* spp. 1QB459	+++	*X*			0.97 ± 0.1	6.90 ± 0.8	0.99
*Levilactobacillus* spp. 2QB383	++		*X*		0.73 ± 0.1	5.46 ± 0.3	0.97
*Levilactobacillus* spp. 3QB167	+	*X*		*X*	1.23 ± 0.0	8.50 ± 0.0	0.99
*Levilactobacillus* spp. 3QB398	+	*X*	*X*		0.70 ± 0.1	5.37 ± 0.5	0.96

α Classification of effect of LAB on growth of *A. parasiticus* in microplate assay. Symbols were assigned according to the effect of each LAB in comparison with the growth of *A. parasiticus* alone (positive control of the fungus). (+++) indicates the maximum effect on growth without total inhibition of the fungus growth by LAB and (+) indicates very little effect of LAB on growth of the fungus.

β All 10 LAB strains were tested individually, in triplicate. Four independent sets of tubes were inoculated, each set related to one of the follow conditions: (1) the LAB strain was inoculated alone (to ensure the ability to grow in YES); (2) the LAB strain was inoculated simultaneously with the fungus (0 h); (3) the LAB strains was inoculated after 24 h incubation of the fungus; and (4) the LAB strains was inoculated after 48 h incubation of the fungus. Incubation was performed at 25°C under static conditions.

γ The symbol *X* was applied when the treatment with the LAB had an effect on growth of the fungus, reducing the mass formed in all three replicates of 10 ml YES broth in test tubes, when compared with the *A. parasiticus* alone (positive control of the fungus). Absence of symbols indicates that no effect of the LAB was shown.

The same level of inoculum was applied to each LAB and the *A. parasiticus* (5.0 log_10_ cfu ml^−1^).

The double layer agar plate was performed by pour-plating 1 ml of LAB inoculum in 10 ml MRS agar, covered by 10 ml YES agar, where 10 μl of the fungus suspension was dispersed on the center of the plate.

Growth rate and maximum diameter of control (*A. parasiticus* alone) were similar to the treatment with *Lactiplantibacillus plantarum* strains 1QB314 and 3QB350.

* The differences shown were not statistically significant at *p* < 0.05.

** Growth diameter of *A. parasitucus* measured on the 7th day of the experiment. The differences shown were not statistically significant at *p* < 0.05.

### Effect of LAB on the Production of Aflatoxins AFB_1_, AFB_2_, AFG_1_, and AFG_2_ by *A. parasiticus*

**Figure 3:**
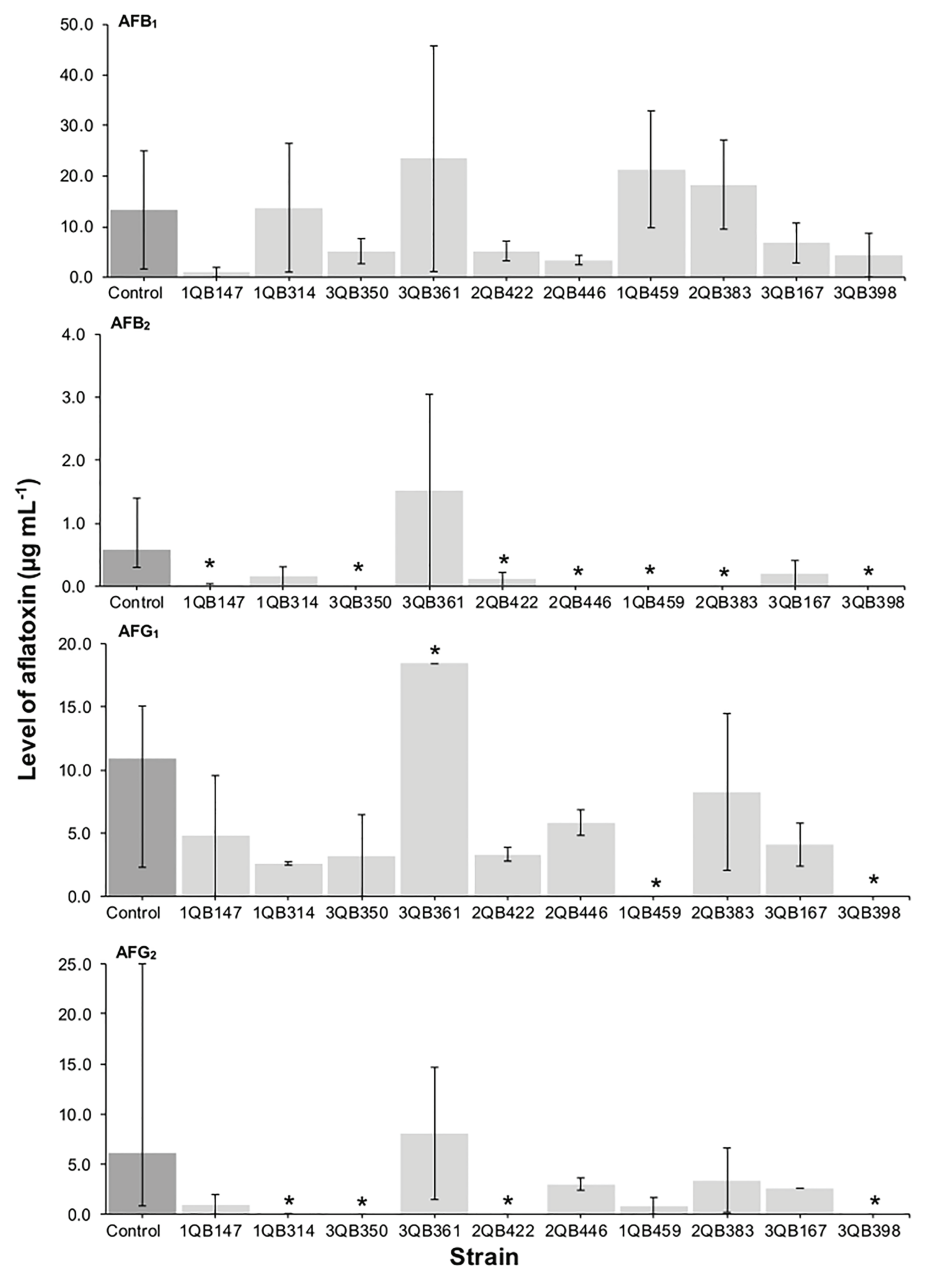
Effect of LAB on production of AFB_1_, AFB_2_, AFG_1_, and AFG_2_ by *A. parasiticus* NRRL 2999 in co-cultured YES broth after 7 days of incubation at 25°C. LAB was inoculated (5 log_10_ cfu ml^−1^) after 24 h (Inoculation = T 24 h) incubation of the fungus. The individually tested LAB included: four strains of *Lactiplantibacillus plantarum* (former *Lactobacillus plantarum*, comprising strains 1QB147, 1QB314, 3QB350, and QB361), two strains of *Levilactobacillus brevis* (former *Lactobacillus brevis*, comprising strains 2QB422 and 3QB446), and four strains of *Levilactobacillus* spp. (former *Lactobacillus* sp., comprising strains 1QB4593, 2QB383, 3QB167, and QB398). Results are expressed as median values of three independent experiments, with minimum and maximum values indicated as error bars. ^*^Median values with asterisks differ significantly from controls (*p* < 0.05).

**Figure 4:**
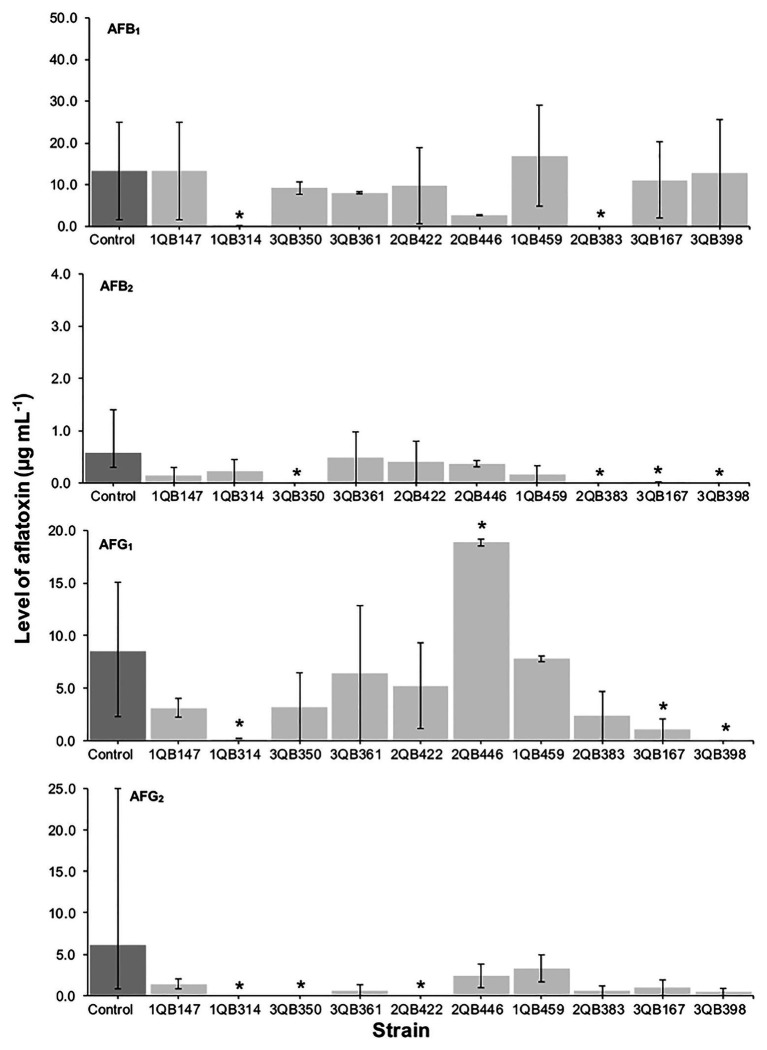
Effect of LAB on production of AFB_1_, AFB_2_, AFG_1_, and AFG_2_ by *A. parasiticus* NRRL 2999 in co-cultured YES broth after 7 days of incubation at 25°C. LAB was inoculated (5 log_10_ cfu ml^−1^) after 48 h (Inoculation = T 48 h) incubation of the fungus. The individually tested LAB included: four strains of *Lactiplantibacillus plantarum* (former *Lactobacillus plantarum*, comprising strains 1QB147, 1QB314, 3QB350, and QB361), two strains of *Levilactobacillus brevis* (former *Lactobacillus brevis*, comprising strains 2QB422 and 3QB446), and four strains of *Levilactobacillus* spp. (former *Lactobacillus* sp., comprising strains 1QB4593, 2QB383, 3QB167, and QB398). Results are expressed as median values of three independent experiments, with minimum and maximum values indicated as error bars. ^*^Median values with asterisks differ significantly from controls (*p* < 0.05).

The effect of the LAB strains on the mycotoxin production by *A. parasiticus* was also investigated in YES broth over 7 days at 25°C. *Aspergillus parasiticus* strains are known to produce AFBs and AFGs, while another *Aspergillus* species commonly found in foodstuffs, *Aspergillus flavus* produce only AFBs (Oliveira et al., 2014). As shown in Figures 1, 3, 4, the moment of inoculation of the LAB strains on the development stage of the fungus, and thus on supressing mycotoxins production was critical. When inoculating the LAB strains at an earlier stage of development of the fungus, nearly all strains reduced the level of the four mycotoxins produced. Greatest inhibition of AFB_1_, AFB_2_, and AFG_2_ production was achieved with strain 3QB350 (*L. plantarum*), followed by 2QB383 (*Levilactobacillus* spp.) and 2QB446 (*L. brevis*). An impressive inhibition ability was shown by 2QB383 (*Levilactobacillus* spp.) toward AFB_1_ (max value of 2.97 μg ml^−1^, while max value of 25.00 μg ml^−1^ was produced by the control without LAB, Figures 1, 4) and AFB_2_ (not detected, Figures 1, 3, 4), and by strain 1QB459 (*Levilactobacillus* spp.) toward AFB_2_ (not detected, Figures 1, 3) and AFG_2_ (max values of 0.41 μg ml^−1^, while max value of 25.00 μg ml^−1^ was produced by the control without LAB, Figure 1). While nearly all strains were able to suppress the production of AFB_2_, only 3QB167 (*Levilactobacillus* spp.), 3QB361 (*L. plantarum*), and 2QB383 (*Levilactobacillus* spp.) were able to inhibit completely the formation of AFG_1_ (Figure 1). Lower effectiveness for strains, as well as larger variation on mycotoxin production was found when they were inoculated 24 h after incubation of the fungus (Figure 1). The varying effect of LAB on mycotoxin production was also reported by Fernández-Juri et al. (2011) when growing *A. parasiticus* NRRL 2999 together with different LAB strains at 25°C for 7 days. These authors reported that AFB_1_ in the control (fungus alone) reached 4.46 ± 0.29 μg g^−1^, but when grown with LAB, the level of AFB_1_ varied between 0.12 ± 0.04 and 7.72 ± 0.56 μg g^−1^, and the variation was dependent of the specific LAB strain. In our study, a similar large difference in the effect of LAB strains on mycotoxin production was found. Mycotoxin production by *A. parasiticus* NRRL 2999 alone (control) was found to vary greatly (dark columns in Figures 1, 3, 4), with median values of 13.31 (AFB_1_), 0.58 (AFB_2_), 19.91 (AFG_1_), and 6.12 μg ml (AFG_2_), but with minimum and maximum values deviating more than 38.1% of the median values (error bars in Figures 1, 3, 4). A 30% variation on mycotoxins production as obtained in our study is considered acceptable due to the intrinsic properties of the fungus (EC, 2002, 2006). This variability is strain-specific and occurs even when the strain is subjected to similar experimental conditions such as performed here (Whiting and Golden, 2002; Freire et al., 2018). Also in agreement with our results, several reports indicate that, once the mycotoxins have reached a maximum production, a decline of its levels occurs, possibly because of the ability of the fungus to degrade or convert the mycotoxins into other metabolites (Freire et al., 2018; Kowalczyk et al., 2019). Furthermore, Greeff-Laubscher et al. (2020) showed that the level of mycotoxin production by a fungus can vary largely within the replicates of a trial as well as length of time of incubation of the fungus, with episodes of levels increasing and decreasing over the whole period. This could explain the increased level of AFs vs. the control for some strains such as 3QB361 (Figure 3) and 2QB446 for AFG_1_ (Figure 4). The largest variation of mycotoxin production was shown in the present study when the LAB strains were inoculated 48 h after the inoculation of the fungus (Figure 4). The number of LAB strains able to inhibit mycotoxin production was lower when the inoculation of the strains was performed after 24 h of the fungus inoculation (Figure 3). The antifungal activity of LAB varies widely according to the species, the incubation conditions (pH, time, and temperature), and the substrate/mycotoxin produced. Generally, the maximum production of anti-fungal compounds occurs at the beginning of the stationary growth phase and decreases over incubation time (Dalié et al., 2010; Gomaa et al., 2018). In a study performed by Sangmanee and Hongpattarakere (2014), *L. plantarum* k35 reached its maximum fungicidal activity at 37°C. This activity was reduced when the strain was incubated at 25°C (temperature at which grains and cereals are generally stored). Strains of *L. brevis* and *L. plantarum* appear to be best aflatoxin inhibitors. Gomaa et al. (2018) investigated a *L. brevis* strain capable of reducing the production of AFB_1_ by 90.4%, and of decreasing the amount of mycelial biomass. In addition to the antifungal effect, bacteria can modify mycotoxins through their enzymatic activity and defense mechanisms and therefore, apparent reduction in the level of mycotoxins is evident. However, the mechanisms of this biotransformation have not yet been elucidated (Freire and Sant’Ana, 2018).

A positive correlation was found between the inhibition of the fungus growth and the inhibition of mycotoxin production (Table 3; Figures 1, 3, 4). An overview of the combined effect of the LAB strains on fungus growth and reduction of aflatoxin production by *A. parasiticus* is shown in Table 4. LAB strains affecting the growth of the fungus, such as strains 2QB422 (*L. brevis*), 2QB383 (*Levilactobacillus* spp), 2QB446 (*L. brevis*), and 3QB398 (*Levilactobacillus* spp.), were also able to inhibit by 50% the production of at least three of the tested aflatoxins. This correlation between inhibition of growth and suppression ability of mycotoxin production by aspergilli was also reported by Ben Taheur et al. (2019b), with a *L. kefiri* strain. Surprisingly, despite no clear inhibition effect of the LAB strains on fungus growth, strong inhibition potential (>50%) on all four aflatoxins was shown by strain 3QB350 (*L. plantarum*), and on at least three of the tested aflatoxins by strains 1QB314 (*L. plantarum*) and 3QB167 (*Levilactobacillus* spp.), at some point of the fungus life cycle (when LAB was inoculated simultaneously with the fungus in Figure 1, when it was inoculated at 24 and 48 h after the fungus was incubated in Figures 3, 4, respectively). This is of particular importance for selection of the best time-point of application of the LAB to obtain the optimum effect and minimize mycotoxin formation in a food matrix. When linked to plant-based foods, the efficiency of strains at earlier stages of the fungus development could be an indication that application on the seeds (instead any other stage of plant development) would be appropriate. Strains more robust and able to affect mycotoxin formation in the later stages of the fungus development, could be an alternative to the application when the fungus is already established in the plant. A similar inhibitory effect of actinomycetes isolates on aflatoxins B1 and B2, but with limited impact on growth of *A. flavus* has been reported (Verheecke et al., 2014). Effect of pomegranate peel extract on AFB_1_ production, but without interfering on fungus growth, was shown by Sadhasivam et al. (2019) when investigating the growth and mycotoxin-producing ability of *A. flavus*. This indicates the relevance of applying selected LAB strains as biological control agents in order to prevent aflatoxin formation. Understanding the mechanisms of this inhibitory effect could be used to optimize the conditions for application purpose.

**Table 4:** Overview of effect of LAB on inhibition of mycotoxigenic *A. parasiticus* NRRL 2999 growth and aflatoxins (AF) production (AFB_1_, AFB_2_, AFG_1_, and AFG_2_), investigated in double layer agar plates with MRS and YES agars and in YES broth, respectively.

LAB strains	Inhibition effect on												
	Growth of fungus inoculated simultaneously with LAB in double layer in MRS-YES agar^α^	Aflatoxin production of fungus in test tube with YES broth inoculated with LAB at different stages (h) of fungus incubation at 25°C ^β^											
		AFB_1_			AFB_2_			AFG_1_			AFG_2_		
		Moment of inoculation with LAB (h)			Moment of inoculation with LAB (h)			Moment of inoculation with LAB (h)			Moment of inoculation with LAB (h)		
		0	24	48	0	24	48	0	24	48	0	24	48
*Lactiplantibacillus plantarum* 1QB147	+	−	+++	−	+++	+++	+++	+++	+	++	++	+++	+++
*Lactiplantibacillus plantarum* 1QB314	−	−	−	+++	−	+++	++	+	+++	+++	+++	+++	+++
*Lactiplantibacillus plantarum* 3QB350	−	+++	+	+	+++	+++	+++	+++	+	+	+	+++	+++
*Lactiplantibacillus plantarum* 3QB361	++	+	−	+	+++	−	+	+++	−	−	+++	−	+++
*Levilactobacillus brevis* 2QB422	+++	+	+	−	+++	+++	+	+++	+++	+	+++	+++	+++
*Levilactobacillus brevis* 2QB446	++	+	++	++	+++	+++	++	−	+	−	+++	+++	++
*Levilactobacillus* spp. 1QB459	+	−	−	−	+++	+++	+++	+++	+++	+	+++	++	+
*Levilactobacillus* spp. 2QB383	++	++	−	+++	+++	+++	+++	+++	−	++	++	+	+++
*Levilactobacillus*spp. 3QB167	−	+	+	−	+++	+++	+++	+++	+	+++	+++	+++	+++
*Levilactobacillus* spp. 3QB398	++	−	+	−	+++	+++	+++	+++	+++	+++	+++	+++	+++

α Classification of effect of LAB on growth of *A. parasiticus*, based on growth rate in Table 3 (μmax) indicating increase (cm day^−1^ during 7 days) values of < 0.70 (+++), 0.70–0.73 (++), 0.97–1.00 (+), or ≥ 1.2(−) in case of no effect of LAB.

β Classification of effect of LAB on production of aflatoxins by *A. parasiticus*, based on potential of reducing more than 50% (+++), equal to 50% (++), less than 50% (+), or without effect (−), when compared the maximum remaining values in Figures 1, 3, 4 with the averaged value for *A. parasiticus* alone (positive control of the fungus, dashed line in Figures 1, 3, 4). LAB was inoculated simultaneously (0 h) or after 24 or 48 h incubation of the fungus at 25°C. Sampling took place after a total of 7 days incubation of the fungus.

The same level of inoculum was applied to each LAB and the *A. parasiticus* (5.0 log_10_ cfu ml^−1^).

The double layer agar plate was performed by pour-plating 1 ml of LAB inoculum in 10 ml MRS agar, covered by 10 ml YES agar, where 10 μl of the fungus suspension was dispersed on the center of the plate.

### Effect of LAB on Reduction of Aflatoxin M_1_ in Spiked Milk

**Figure 5:**
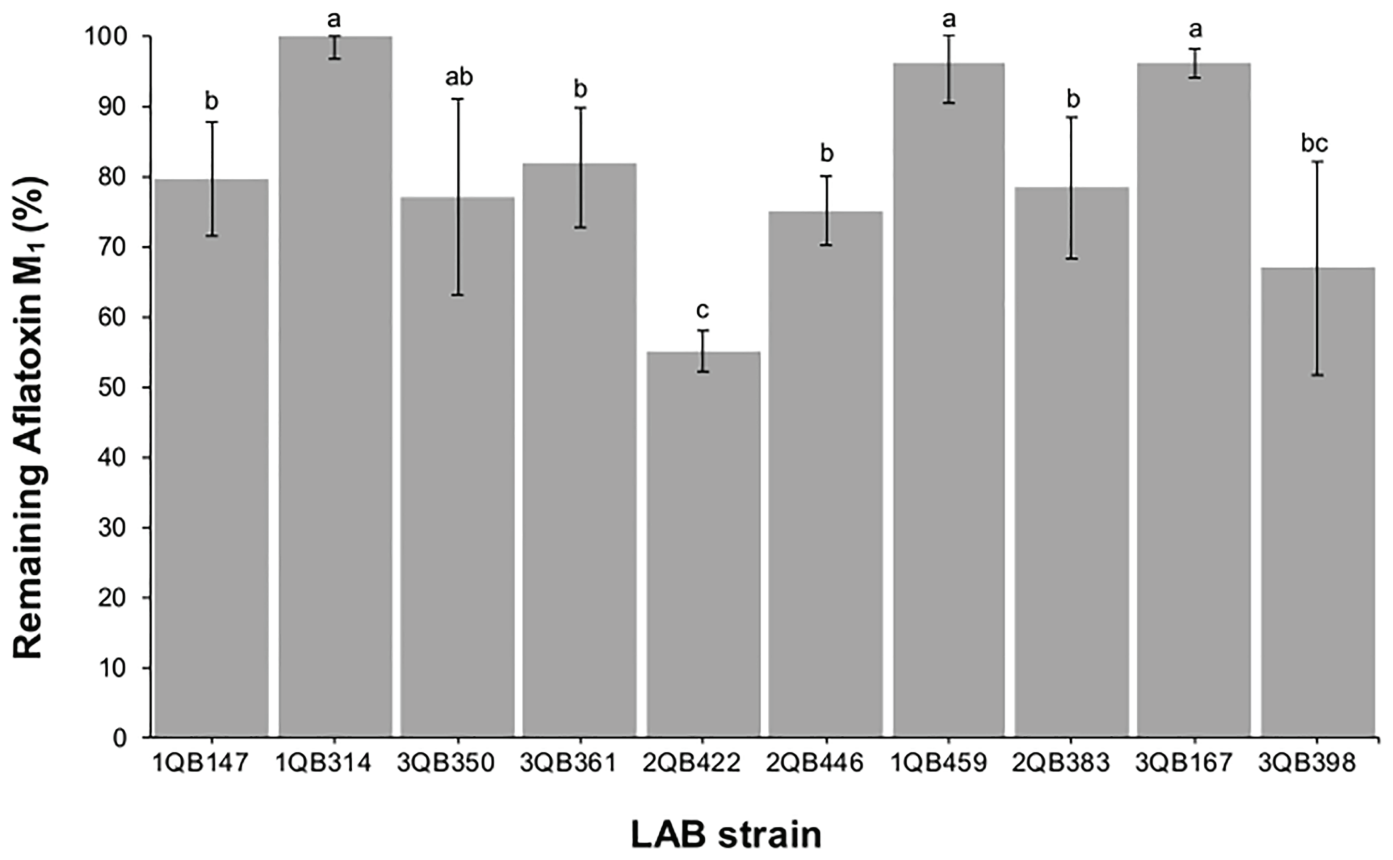
Remaining aflatoxin M_1_ in skimmed milk samples spiked at 0.5 μg ml^−1^, after 15 min contact time with heat-inactivated cells of individually tested LAB including: four strains of *Lactiplantibacillus plantarum* (former *Lactobacillus plantarum*, comprising strains 1QB147, 1QB314, 3QB350, and QB361), two strains of *Levilactobacillus brevis* (former *Lactobacillus brevis*, comprising strains 2QB422 and 3QB446), and four strains of *Levilactobacillus* spp. (former *Lactobacillus* sp., comprising strains 1QB4593, 2QB383, 3QB167, and QB398). Results (% in relation to controls) are expressed as mean values of three independent experiments, with SDs indicated as error bars. Bars with no common letters differ significantly (*p* < 0.05).

After treatment with the heat-inactivated LAB cells, the remaining levels of mycotoxins in spiked milk samples (Figure 5) indicated that a small reduction (about 18–25%) was achieved with strains 3QB361 (*L. plantarum*), 1QB147 (*L. plantarum*), 2QB293 (*Levilactobacillus* spp.), 3QB350 (*L. plantarum*), and 2QB446 (*L. brevis*). Greater reductions of 33 and 45% were achieved by treatment with strains 3QB398 (*Levilactobacillus* spp.) and 2QB422 (*L. brevis*), respectively. Our results were similar to those of Bovo et al. (2013), who under similar conditions, reported reductions ranging from 12.42 to 45.67% for inactivated cells and between 5.60 and 33.54% for viable cells. In a study performed by Hashemi and Gholamhosseinpour (2019), all strains of *Levilactobacillus* spp. reduced the amount of AFM_1_ by 26–52% in fermented cream. Mycotoxin binding with LAB cells is essentially a surface phenomenon, which involves non-covalent bonds between the cell wall components and the mycotoxin dissolved in the liquid medium (Haskard et al., 2001). Kuharić et al. (2018) selected, isolated, and identified 10 LAB species from raw milk with the objective to select a strain to efficiently bind AFM_1_. In addition, they observed a binding efficiency ranging from 21 to 92% for viable cells, and from 26 to 95% for thermally treated cells. Similar to our results, Corassin et al. (2013) observed that a pool of heat-killed cells of LAB strains (*Lb. rhamnosus*, *Lb. delbrueckii* spp. *bulgaricus*, and *Bifidobacterium lactis*) bound 11.5–11.7% of AFM_1_ in ultra-high temperature skim milk after 30–60 min. The binding percentages of heat-killed cells of *Lb. bulgaricus*, *Lb. rhamnosus*, and *B. lactis* to AFM_1_ in UHT skimmed milk were 13.5, 19.7, and 37.7%, respectively (Bovo et al., 2013), which are in range of the values described in the present work.

### Effect of LAB on Reduction of AFB_1_, OTA, and ZEN in Buffer

**Figure 6:**
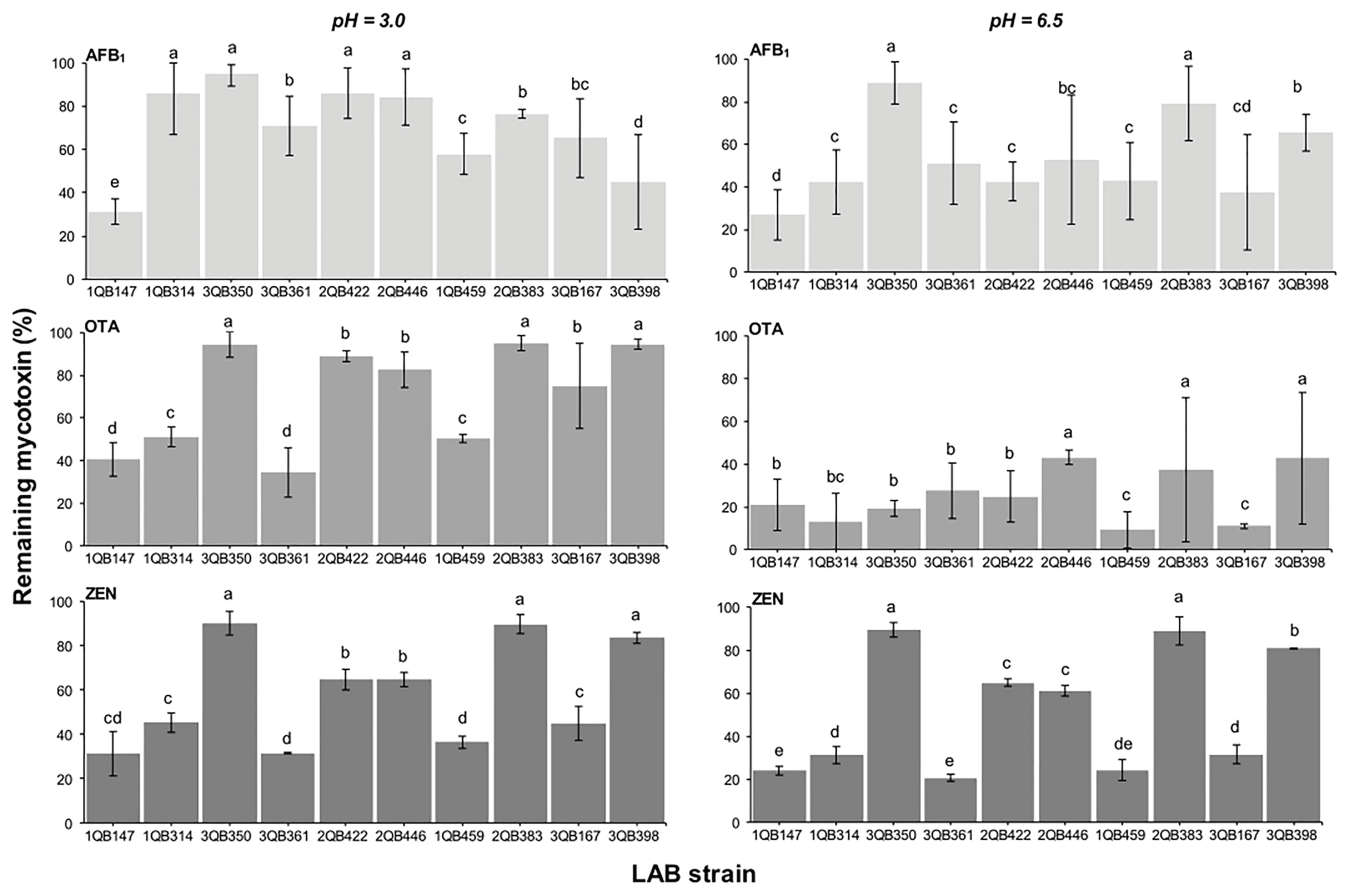
Remaining AFB_1_, OTA, and ZEN in PPB at pH 3.0 and 6.5, after 15 min contact time with heat-inactivated LAB strains including: four strains of *Lactiplantibacillus plantarum* (former *Lactobacillus plantarum*, comprising strains 1QB147, 1QB314, 3QB350, and QB361), two strains of *Levilactobacillus brevis* (former *Lactobacillus brevis*, comprising strains 2QB422 and 3QB446), and four strains of *Levilactobacillus* spp. (former *Lactobacillus* sp., comprising strains 1QB4593, 2QB383, 3QB167, and QB398). Initial concentrations of mycotoxins in PPB were 1.0 ng ml^−1^ (AFB_1_, OTA) and 2.0 μg ml^−1^ (ZEN). Results (% in relation to controls) are expressed as mean values of three independent experiments, with SDs indicated as error bars. Bars with no common letters differ significantly (*p* < 0.05).

**Figure 7:**
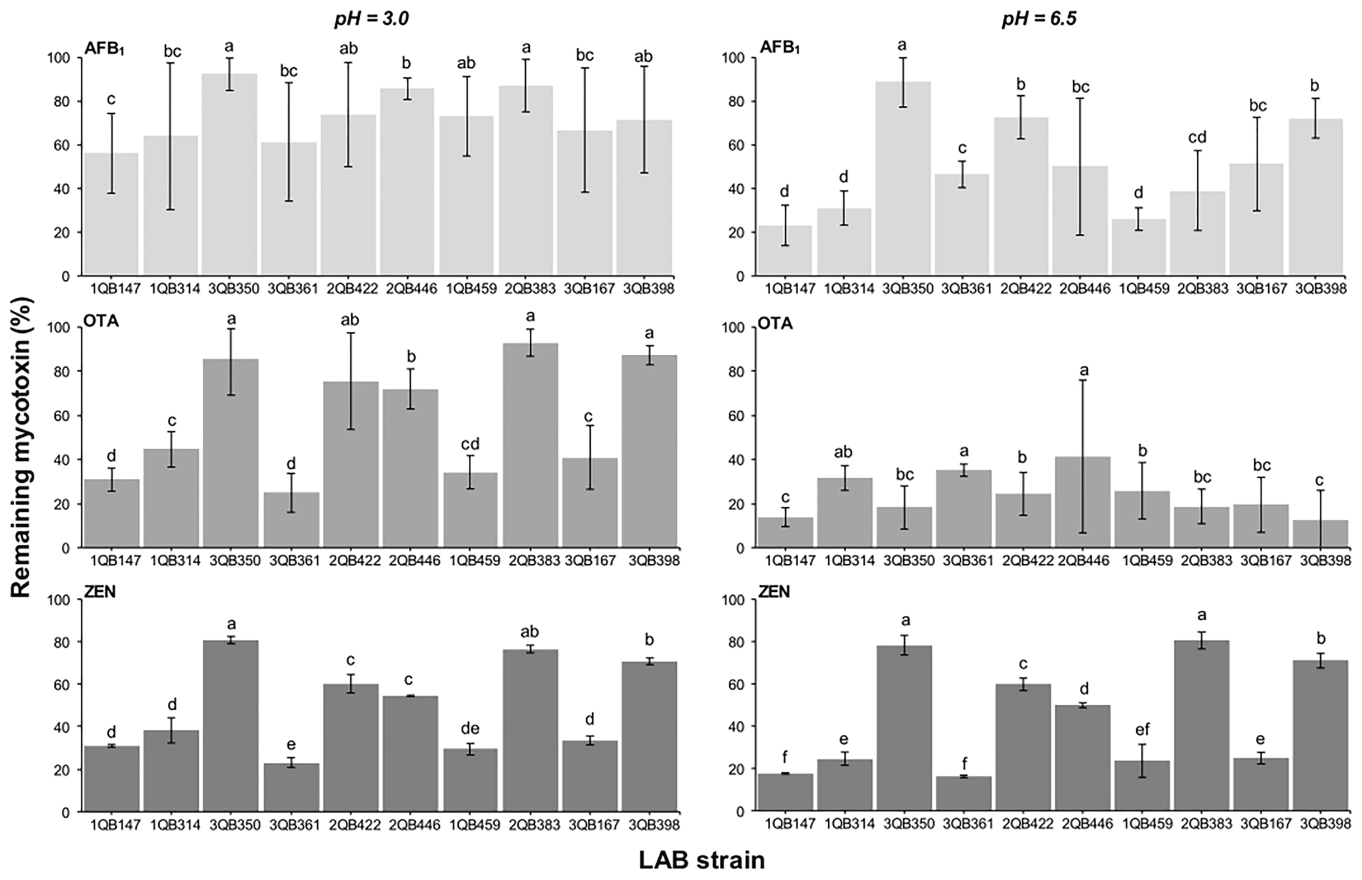
Remaining AFB_1_, OTA, and ZEN in PPB at pH 3.0 and 6.5, after 5 min contact time with heat-inactivated LAB strains including: four strains of *Lactiplantibacillus plantarum* (former *Lactobacillus plantarum*, comprising strains 1QB147, 1QB314, 3QB350, and QB361), two strains of *Levilactobacillus brevis* (former *Lactobacillus brevis*, comprising strains 2QB422 and 3QB446), and four strains of *Levilactobacillus* spp. (former *Lactobacillus* sp., comprising strains 1QB4593, 2QB383, 3QB167, and QB398). Initial concentrations of mycotoxins in PPB were 1.0 μg ml^−1^ (AFB_1_, OTA) and 2.0 μg ml^−1^ (ZEN). Results (% in relation to controls) are expressed as mean values of three independent experiments, with SDs indicated as error bars. Bars with no common letters differ significantly (*p* < 0.05).

When testing the effect of LAB strains on the reduction of AFB_1_, OTA, and ZEN in spiked buffer, the effect of time of exposure with the inactivated LAB cells was important for the robustness of the reduction. The mycotoxin decline was more robust with less variation when the spiked buffer was treated for 15 min (Figure 6), in comparison to a treatment for 5 min (Figure 7). The incubation period was chosen based on the experiments described by Bovo et al. (2013), who showed that the viable and heat-killed cells of LAB have the same ability to bind to AFM_1_ in 15 min and 24 h of contact. In addition, a recent review by Muaz et al. (2021) indicated that the process of decontamination is a rapid phenomenon during which the binding occurs within the first few minutes, so a longer incubation time may not result in any significant difference among decontamination levels. In addition to the effect of time of exposure, the reduction of the mycotoxins was greatest at pH 6.5 (D, E, and F in Figures 6, 7). The LAB strain 1QB147 (*L. plantarum*) was the most effective in reducing the level of AFB_1_ (Figures 6, 7), independent of the pH, and duration of treatment. LAB strain 3QB361 (*L. plantarum*) was the most effective strain in reducing the levels of OTA (Figures 6, 7) at pH 3.0 (independent of the duration of treatment). LAB strains 3QB398 (*Levilactobacillus* spp.) and 1QB459 (*Levilactobacillus* spp.) resulted in a greater decline of OTA at pH 6.5 when treated for 5 (Figure 7) and 15 min (Figure 6), respectively. Regardless of the pH and duration of treatment, strain 3QB361 (*L. plantarum*) had the highest impact on reducing ZEN (C and F in Figures 6, 7), followed by strains 1QB147 (*L. plantarum*) and 1QB459 (*Levilactobacillus* spp.). The high mycotoxin binding efficiencies of some LAB strains evaluated in the present study highlight their potential use as additives for reducing the bioavailability of mycotoxins in foods. In this context, LAB have been tested for reducing mycotoxins in foods, such as artificially contaminated almonds and peanuts, with *L. kefiri* FR7 able to decrease by 85.3% the level of AFB_1_ and by 83.9% the level of AFB_2_ in almonds, in addition to reducing by 25% OTA in peanuts (Ben Taheur et al., 2019b). Ben Taheur et al. (2017) demonstrated a high mycotoxin binding capacity of Kefir-originated strains of *L. kefiri*, *Kazachstania servazziia*, and *Acetobacter syzygii*, with *L. kefiri* KFLM3 reducing approximately 82% of ABF_1_, 94% of OTA, and 100% of ZEN in milk. In addition, another study demonstrated that strains of *L. plantarum* in PPB and MRS medium were efficient in decreasing ZEN after 48 h of incubation (Zhao et al., 2015). ZEN reduction of up to 30% through adsorption was observed with *L. lactis* (Rogowska et al., 2019). Thus, taking together, our findings and the previous reports indicate that LAB may be a potential tool for reducing mycotoxins in foods.

The removal of mycotoxins by LAB usually occurs by adsorption (Bueno et al., 2007), in which mycotoxin molecules bind reversibly to the structures present in the bacterial cell wall (peptidoglycans, carbohydrates, polysaccharides, and teichoic and lipoteichoic acids) through ion exchange, complexation, and hydrophobic iterations (Dalié et al., 2010; Ben Taheur et al., 2017; Afshar et al., 2020). Because it is a physical phenomenon, most chemical and physical treatments (e.g., application of heat) intended to kill cells do not affect this property and, in some cases, even increase its performance, due to the exposure of the binding sites (Dalié et al., 2010). Another factor that can contribute to the reduction of aflatoxins by adsorption is the production of exopolysaccharides (Ben Taheur et al., 2017), a characteristic present in three of the four LAB mentioned above (*Levilactobacillus* spp. 1QB459, *L. plantarum* 3QB361, and *Levilactobacillus* spp. 3QB398; see supplementary material in Margalho et al., 2020).

## Conclusion

From the 10 LAB strains tested in the present study, all had an effect on mycotoxins. Some strains suppressed the growth and/or aflatoxins (AFB_1_, AFB_2_, AFG_1_, and AFG_2_) production ability of *A. parasiticus*, to different degrees. Some strains were able to bind with mycotoxins, such as AFB_1_, OTA, and ZEN, at different levels of efficiency, which was influenced by the applied conditions (pH and time of treatment), as well as the mycotoxin and the matrix used (milk or buffer). The findings of the present study are very relevant, especially considering the critical toxic effect of mycotoxins as well as the increasing mycotoxin occurrence worldwide. The purpose of screening for LAB with the ability to minimize mycotoxins in milk and *in vitro* models was demonstrated. Despite challenging and time consuming, applicability and efficiency of these LAB in different food matrices are worthy of investigation. It is important to consider that the LAB strains tested in the present study were isolated from cheese, and they have the ability to control aflatoxins in foodstuff such as milk, and this may be considered as an asset for the development of integrated management systems for aflatoxin reduction in cheese production. The results clearly indicate the promising potential of LAB in reducing the risk related to mycotoxins. This potential could even be explored further for the safer production of plant-based foods.

## Data Availability Statement

The original contributions presented in the study are included in the article/supplementary material; further inquiries can be directed to the corresponding author.

## Author Contributions

CM designed the experimental work as well as acquired and analyzed the data, and wrote and reviewed the manuscript. LF contributed by acquiring and modeling the data from the growth experiments, and reviewing the manuscript. RR acquired the data using LC-LC/MS. LM, CB, and LTF acquired the data and reviewed the manuscript. AS, FR, and CC supervised the work in their respective labs, and reviewed the manuscript. CO conceptualized the work, supervised the project, interpreted the data, and reviewed the manuscript. All authors contributed to the article and approved the submitted version.

### Conflict of Interest

The authors declare that the research was conducted in the absence of any commercial or financial relationships that could be construed as a potential conflict of interest.
